# Intelligent Sensors for Human Motion Analysis

**DOI:** 10.3390/s22134952

**Published:** 2022-06-30

**Authors:** Tomasz Krzeszowski, Adam Switonski, Michal Kepski, Carlos T. Calafate

**Affiliations:** 1 Faculty of Electrical and Computer Engineering, Rzeszow University of Technology, 35-959 Rzeszow, Poland; 2 Department of Graphics, Computer Vision and Digital Systems, Silesian University of Technology, 44-100 Gliwice, Poland; adam.switonski@polsl.pl; 3 Institute of Computer Science, University of Rzeszów, 35-310 Rzeszow, Poland; mkepski@ur.edu.pl; 4 Computer Engineering Department, Universitat Politècnica de València (UPV), 46022 Valencia, Spain; calafate@disca.upv.es

Currently, the analysis of human motion is one of the most interesting and active research topics in computer science, especially in computer vision. The great interest in this area is due to the wide range of promising applications in many fields, such as medicine, surveillance systems, sports performance analysis, virtual reality, human–computer interaction, etc. Human motion analysis concerns the detection, tracking, and recognition of people and their activities based on data recorded by various types of sensors. In these studies, RGB and depth cameras are often used. Additionally, research aimed at developing gait and action recognition methods often uses motion capture systems based on active or passive markers and IMU sensors. These systems are challenging to develop, but also offer possibilities to solve advanced research problems, especially when only visual data are used. Other types of sensors that are used in motion analysis are pressure platforms and EMG sensors.

The Special Issue (SI) entitled “Intelligent Sensors for Human Motion Analysis” focuses on many aspects of human motion analysis. The Issue raised concerns of, among others, pose estimation, action and gait recognition, fall detection, as well as EMG signal processing, pressure platform construction, and issues related to improving motion capture acquisition.

As mentioned, the analysis of human movement is an important and extensive research problem, with many potential applications. This is the subject of the paper [1], which focuses on a review of the applications of pose estimation in human health and performance throughout life. The authors provided many examples of the usage of this type of system, but focused specifically on applications in the areas of human development, performance optimization, injury prevention, and motor evaluation of people with neurologic damage or disease. An extensive review of 125 scientific papers includes an overview of available tools, their use in improving human health and performance, and a discussion of the limitations and implementation problems associated with pose estimation. Moreover, the authors anticipate that, despite the existence of many limitations, the applications of pose estimation in human health and performance will continue to expand in the coming years, and that these technologies will provide powerful tools to capture significant aspects of human movement that have been difficult to register using conventional techniques.

The issues related to the estimation of the pose were also discussed in papers [2,3]. In [2], Rapczyński et al. investigated the commonly used datasets, discussed their biases and used them in cross-database experiments. They also proposed a method to harmonize the definitions of skeleton joints specific to the dataset and a scale normalization method that significantly improves generalization across cameras, subjects, and databases by up to 50%. The experiments carried out showed the negative effect of the biases of the dataset on generalization, as well as the positive impact of the proposed method of scale normalization. The authors also investigated the effect of using more or fewer cameras (also virtual cameras), training with multiple datasets, and using the OpenPose library.

The more difficult challenge is to estimate the pose using a monocular camera. In [3], a real-time framework is proposed for the estimation of 3D absolute poses of multiple people using a monocular camera. The developed system, called Root-GAST-Net, combines a human detector, a 2D pose estimator, a 3D root-relative pose reconstructor, and a root depth estimator in a top-down approach. The framework is based on modified versions of the RootNet and GAST-Net networks. In the experiments, the proposed method outperformed the state-of-the-art method. Furthermore, real-time processing was achieved using the Nvidia GeForce GTX 1080.

Another research area that has been widely studied is the problem of gait recognition. In [4], hybrid methods are proposed that combine regularized discriminant analysis (RDA) and swarm intelligence techniques for gait recognition. These techniques are utilized to tune the observation weights and hyperparameters of the RDA method to minimize the objective function. In the investigation, three well-known optimization algorithms were used: particle swarm optimization (PSO), grey wolf optimization (GWO), and whale optimization algorithm (WOA). The experiments carried out confirmed the usefulness of the developed methods.

In turn, Moro et al. [5] presented an approach for markerless gait analysis based on RGB video data and deep learning algorithms. To detect 2D feature points in the image, the AdaFuse algorithm was used. Then, the acquired 2D points were used to determine 3D points and generate the human biomechanical skeleton models. The results obtained by the proposed method were compared with the data registered by the marker motion capture system.

Data augmentation is an important technique in machine learning, focusing on the enhancement of the size and quality of training datasets. In [6], a new method for action recognition time series augmentation is introduced. The method determines constraints on the generated data using statistics for a class and its representatives. The method has been compared with other approaches on eight datasets from the action recognition domain.

Recognizing and monitoring activities of daily living are an important part of understanding human behavior. Several approaches emerged to distinguish between activities of daily living and falls, focusing mainly on camera-based and inertial measurements. Some techniques analyze not only a person’s movement, but also their static pose, the correct recognition of which can carry important cues for fall detection. In [7], the recognition of the lying pose from a depth map is approached with a new hybrid FRSystem. Due to the application of the LEM2 algorithm, it was possible to reduce the number of rules almost twofold, making the inference system more interpretable by a human expert.

Detection is not the only research topic related to human falls another important issue is the assessment of physical function and the risk of falls. An automated system proposed in [8] predicts the patients’ score on the well-known Berg Balance Scale (BBS) using motion data captured by a multiple camera system. Furthermore, machine learning methods were used to develop fall risk predictors that reduce the number of tasks required to assess fall risk, without compromising the accuracy of the classic BBS assessment.

Human motion analysis may be applied not only to individuals, but also to groups and gatherings. In [9], different depth sensors (Kinect v2, Azure Kinect, and Zed 2) were evaluated in terms of accuracy to assess body orientation angles to detect spaces occupied by social groups using the F-Formations model. In addition, the advantages and disadvantages per device in determining the body orientation were discussed, and an experimental setup for such tasks was presented.

In [10], a deep learning approach is proposed for the human action recognition problem, utilizing existing architectures and transfer learning. The solution consists of multiple steps, including feature mapping, feature fusion, and feature selection. Deep features are fused using the Serial-based Extended (SbE) approach, and the best features are selected using kurtosis-controlled weighted KNN.

In [11], the authors proposed a non-contact monitoring and classification system for breathing patterns using the XGBoost classifier and Mel-frequency cepstral coefficient (MFCC) feature extraction. Breathing patterns are observed using FMCW radar technology that can be used to develop non-contact medical devices. The authors discuss data analysis, as well as the detailed implementation of hardware-based signal processing. The results of the respiratory pattern classification were presented on a dataset consisting of 4000 samples imitating five breathing patterns, where an 87.375% accuracy was achieved.

The most precise measurements of human movements are provided by optical motion capture systems. The acquisition is based on the calibrated multicamera setup that tracks the 3D coordinates of markers attached to the human body. Although the registration accuracy is high, motion capture systems are not error-free. In fact, occlusions can cause markers to become undetectable. The time instants of a motion sequence with missing markers are called gaps. They require some kind of post-processing to reconstruct missing data, a process that can be performed manually by humans. However, it is a time-consuming operation, and it can be completed only by the experienced and skilled staff of a motion capture laboratory. Thus, automatic methods of gap reconstruction are highly demanded. In [12], feed-forward neural networks, three variants of recurrent networks (gated recurrent unit, long-short-term memory, and bidirectional LSTM), and interpolation techniques (linear, spline, modified Akima, piecewise cubic Hermite, and polynomial), as well as low-rank matrix completion techniques, are employed to predict trajectories of the lost markers.

The applied reconstruction techniques for mocap data and acquisition noise can result in another issue—momentary systematic errors called artefact distortion. They introduce trajectory modifications of different types and scales. In [13], four existing types of artefacts are detected, classified, and removed. The proposed algorithm is based on the derivative, low-pass filtering, mathematical morphology, loose predictor, and applicability analysis. In the validation, multiple simulations using synthetically distorted sequences are used. The outcomes are compared to human performance in the detection and removal of artefact distortion.

The optical marker-based motion capture acquisition has serious limitations as regards its practical applications. The multicamera system has to be mounted in the laboratory and calibrated prior to being used. Moreover, markers are attached to the human body before registration. Thus, the research on the effective markerless acquisition of motion data is of great importance. In [14], the challenge of three-dimensional human mesh reconstruction from a single video is faced. The human pose refinement network based on a non-local attention mechanism is applied to refine the noisy sequence of 3D human poses. It consistently improves the performance of existing state-of-the-art methods.

Another widely studied research area is the problem of recognition of facial emotions, which are expressed by human mimicry. In [15], grammatical facial expressions especially important for sign languages are recognized. The proposed approach extracts time sequences containing selected action units and facial landmarks using the OpenFace library, and classifies them by the chosen deep neural networks. Another contribution of the paper is related to the collected LSE_GFE dataset. It contains isolated signs, expressive sentences, interviews, and annotations for some grammatical facial expressions.

Human motion can also be described by EMG data. They describe the electrical activities of muscles in successive time instants. In [16], the human–machine interface based on the EMG registration is designed and successfully applied to control the robotic manipulator. The interface utilizes a multilayer neural network that identifies four different classes of muscle contraction, and a state machine for the transition change of the manipulator.

In another variant, motion is represented by the ground reaction forces. They describe the reaction of the ground to the body in contact. In [17], a low-cost wearable insole unit is developed that measures plantar pressure. It is based on the principle of photoelectric sensing and performs measurements for six selected key points of the human foot.

The SI entitled “Intelligent Sensors for Human Motion Analysis” comprises 17 articles on numerous aspects related to human motion analysis, which were briefly overviewed above. New techniques and methods for pose estimation, gait recognition, and fall detection have been proposed and verified. Some of them will trigger further research, and some may become the backbone of commercial systems.

It can be noticed that human motion analysis and related matters are challenging and important hot topics. There are still a lot of issues to be addressed, and so an exciting future is expected for this research area.
